# A Literature Review on Pain Management in Women During Medical Procedures: Gaps, Challenges, and Recommendations

**DOI:** 10.3390/medicina61081352

**Published:** 2025-07-26

**Authors:** Keren Grinberg, Yael Sela

**Affiliations:** Nursing Sciences Department, Faculty of Social and Community Sciences, Ruppin Academic Center, Emek Hefer 4025000, Israel

**Keywords:** pain management, women’s health, gynecological procedures, gender disparities, analgesia, healthcare equity

## Abstract

*Background and Objectives*: Gender disparities in pain management persist, with women frequently receiving inadequate analgesia despite reporting similar or higher pain levels compared with men. This issue is particularly evident across various medical and gynecological procedures. *Materials and Methods*: This integrative literature review synthesizes recent empirical studies examining gender biases in pain perception and management, focusing specifically on procedural pain in women. It includes an analysis of clinical research, patient-reported outcomes, and healthcare provider behaviors. *Results*: The findings indicate that unconscious biases, a lack of gender-specific clinical protocols, and prevailing cultural stereotypes contribute to the undertreatment of pain in women during procedures such as intrauterine device insertion and diagnostic hysteroscopy. Additionally, communication gaps between patients and healthcare providers exacerbate these disparities. *Conclusions*: Addressing gender disparities in pain management necessitates systemic reforms, including the implementation of gender-sensitive clinical guidelines, enhanced provider education, and targeted policy changes. Personalized, gender-informed approaches are essential to improving equity and quality of care in pain treatment.

## 1. Background

Pain is a fundamental biological and emotional experience that transcends age, geography, and culture. It plays a critical protective role, alerting the body to potential or actual harm [1]. Despite its centrality to human health, pain assessment and management remain complex due to the subjective nature of pain perception. Among the most pressing issues in contemporary medicine is the persistent gender gap in pain recognition and treatment. Inadequate pain management for women is a documented issue in healthcare, with studies showing that women are less likely to receive adequate pain relief compared with men, even when reporting similar pain levels. Women are more likely to have their pain underestimated, misdiagnosed, or undertreated across various healthcare settings [2,3].

Despite decades of progress in medical science, women continue to encounter systematic challenges in receiving timely and effective pain care. Research has demonstrated that women often face underdiagnosis and inadequate treatment for their pain, a trend observed across multiple healthcare environments [4].

Numerous studies have documented significant differences in how men and women experience, report, and are treated for pain. Hormonal fluctuations, such as variations in estrogen and progesterone, are known to influence pain thresholds and sensitivity in women [5]. Psychological and sociocultural factors also play a substantial role in how individuals express pain and how their symptoms are perceived by healthcare providers. Men are often treated with faster and more targeted pharmacological interventions, including opioids, whereas women’s pain complaints are frequently attributed to psychosocial causes such as anxiety or depression [6,7,8,9]. This leads to delayed diagnosis and treatment and often results in suboptimal care.

Recent studies further emphasize these disparities. For instance, a 2024 study demonstrated that female patients presenting to emergency departments were significantly less likely than male patients to receive analgesic medications—both opioid and non-opioid—even when reporting equivalent pain scores [10]. Additionally, foundational research has shown that postoperative pain in women was often managed with sedatives rather than appropriate analgesics, whereas men received more consistent pain medication [11]. These differences in treatment practices are supported by experimental evidence showing physiological and behavioral differences in how pain and analgesia are processed according to gender, emphasizing that the disparities are not solely due to reporting bias but also to systemic misinterpretation and outdated assumptions about pain perception [12].

Gender disparities in pain management are particularly evident in emergency departments, surgical settings, and gynecological care, where women often receive delayed or insufficient analgesia despite reporting comparable or higher pain levels than men [13,14,15]. In acute medical scenarios, such as cardiovascular events, gender bias can have critical implications. Women with chest pain often experience atypical symptoms and face longer delays in evaluation. Studies have revealed that they are less likely to receive timely ECGs, are more frequently transported to hospitals lacking advanced cardiac intervention capabilities, and generally have poorer outcomes than men [16].

Beyond acute care, women are disproportionately affected by chronic pain conditions, including endometriosis, fibromyalgia, interstitial cystitis, and vulvodynia—conditions that are frequently underdiagnosed and underfunded [17,18]. These chronic conditions contribute significantly to long-term disability, diminished quality of life, and psychological strain. Despite their prevalence and severity, research funding and clinical awareness for these disorders remain insufficient.

The roots of these disparities can be traced to long-standing gender biases embedded in medical history and education. Historically, women have been portrayed in clinical discourse as more emotional or exaggerative, which has contributed to a systematic underestimation of their symptoms. Such stereotypes persist in subtle forms in modern clinical practice, shaping diagnostic and treatment decisions, often without conscious intent [19].

Recent public health data underscore the extent of gender disparities in pain care. According to the 2022 Gender Pain Gap Index Report, over 56% of women felt their pain was dismissed by healthcare providers, and nearly one-third avoided or delayed care due to concerns about not being taken seriously [20]. These findings highlight the urgent need for systemic reform in clinical training and healthcare policy.

Certain routine medical procedures disproportionately affect women and are associated with poor pain management practices. For example, intrauterine device (IUD) insertions and hysteroscopies are frequently conducted without adequate analgesia, despite consistent reports from patients of significant pain during these procedures [18,19]. Similarly, women undergoing urological or colorectal interventions often receive limited preprocedural counseling about potential pain or available pain control options, reflecting a broader pattern of inadequate preparation and support. Many women also report experiencing moderate to severe pain during routine procedures such as cervical biopsies and pelvic exams without being offered appropriate analgesia or sedation. Even when effective pain relief protocols exist, they are inconsistently applied across clinical settings, contributing to avoidable discomfort and distress [21].

The aim of this literature review is to present current research findings regarding pain management in women undergoing various medical procedures, examine the underlying causes of existing disparities, and explore ways to improve pain treatment in healthcare. The review focuses on identifying contributing factors, clinical and experiential outcomes of inadequate pain management, and emerging solutions within the healthcare system. It synthesizes empirical findings from clinical, qualitative, and systematic review studies published over the past decade to provide a comprehensive overview of the challenges and opportunities for change. The rationale for this review stems from the urgent need to promote evidence-based, gender-sensitive pain management and address systemic biases in medicine. Improving pain care for women is an ethical, professional, and public health priority requiring a deep understanding of the problem’s scope and effective responses.

## 2. Methods

This structured integrative literature review was conducted to identify and synthesize empirical studies published between 2014 and 2024 that address the undertreatment of pain in women during medical procedures. The review followed a systematic search strategy and predefined inclusion and exclusion criteria to ensure transparency and comprehensiveness. A comprehensive literature search was conducted in four major scientific databases: PubMed, Scopus, Web of Science, and CINAHL. The search terms included combinations of keywords such as “pain management,” “gender differences,” “undertreatment of pain,” “women and pain,” “medical procedures,” “analgesia disparities,” and “implicit bias in healthcare.” Articles published in English and Hebrew were considered. Studies were included if they (1) were published between 2014 and 2024, (2) examined pain management in women undergoing medical procedures, (3) provided a gender-based analysis of treatment outcomes, and (4) included quantitative clinical indicators (e.g., medication use, pain scores) or qualitative data (e.g., patient narratives). Studies focusing exclusively on pediatric populations, chronic pain only, or articles lacking empirical data (e.g., opinion pieces or theoretical essays) were excluded. Screening was conducted in two stages by two independent reviewers. Titles and abstracts were first screened for relevance, followed by a full-text review of potentially eligible articles. Any discrepancies were resolved through discussion until consensus was reached.

The methodological quality of included studies was assessed using appropriate critical appraisal tools based on study design (e.g., CASP for qualitative studies, Newcastle–Ottawa Scale for observational studies). Data extraction included study characteristics, outcomes, and key findings associated with gender disparities in pain management. Given the heterogeneity of study designs and outcome measures, a narrative synthesis approach was used to summarize and interpret the results.

**Figure 1:**
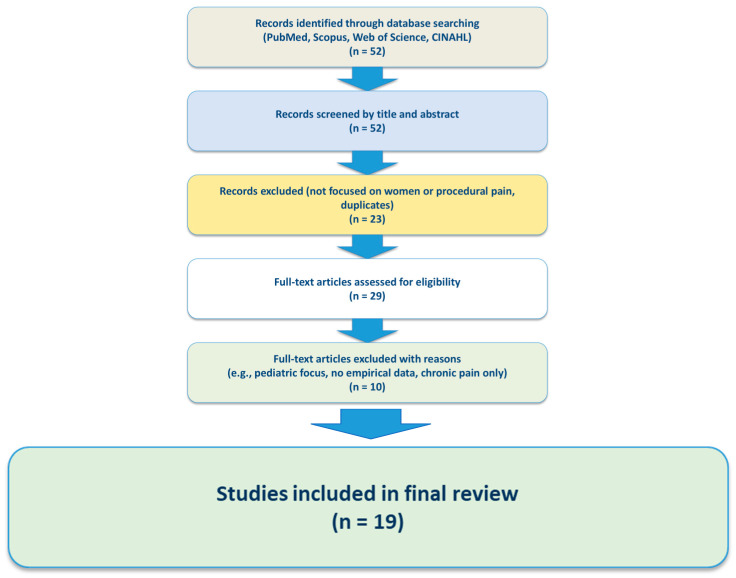
Flow diagram of study selection.

A total of 52 studies were initially identified, of which 19 met the inclusion criteria and were included in the final synthesis. The selected studies included quantitative research, systematic reviews, qualitative interviews, and observational studies, with the majority conducted in hospital-based clinical settings (Figure 1).

The methodological quality of each included study was assessed using appropriate critical appraisal tools tailored to the study design—such as the CASP checklist for qualitative studies and the Newcastle–Ottawa Scale for observational research. Studies were evaluated based on several criteria, including clarity of objectives, sampling methods, control for confounders, and outcome measurement. Although all selected studies met the minimum quality thresholds, there was notable variation in methodological rigor, which was considered during the synthesis and interpretation of results.

## 3. Findings

**Figure 2:**
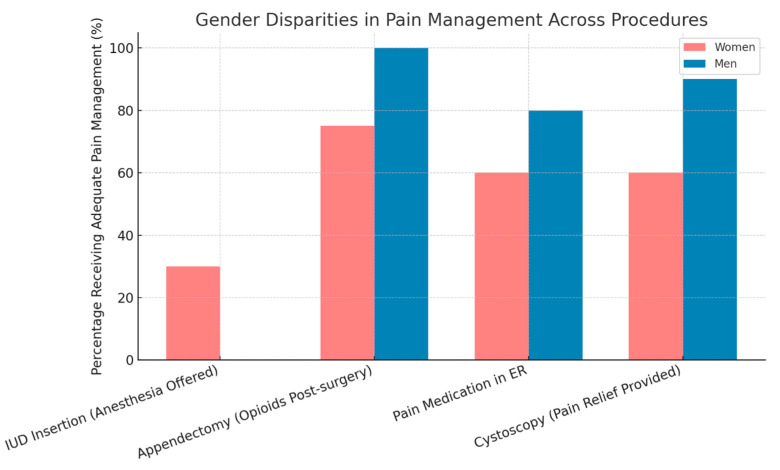
**Gender disparities in pain management across medical procedures.** The chart illustrates the percentage of women and men who received adequate pain management during four procedures: IUD insertion, appendectomy, pain treatment in the emergency room, and cystoscopy. In all cases, men were more likely than women to receive appropriate pain relief, highlighting significant gender gaps in clinical pain management practices.

The findings reveal a consistent pattern of gender disparities in pain management, with many women not receiving adequate treatment, even when their reported pain levels are equal to or higher than those of men (Figure 2). A study by Samulowitz et al. [13] found that women often receive fewer pain medications in emergency departments and outpatient clinics, even when their pain indicators are comparable to those of men; additionally, women undergoing gynecological procedures report more severe pain than what physicians estimate [22].

A notable example of this is the insertion of intrauterine devices (IUDs), where only 30% of physicians offer anesthesia, despite approximately 70% of women reporting moderate to severe pain [23]. Research supports the effectiveness of cervical anesthesia in reducing pain perception during such procedures [24]. Similarly, diagnostic hysteroscopies performed without anesthesia have been associated with pain levels rated between 7 and 9 out of 10, especially among women with anxiety, suggesting the need for a holistic approach that includes psychological support [25].

In other routine procedures, such as cystoscopy, about 40% of clinics provide no pain relief beyond suggesting relaxation techniques, despite frequent reports of burning or sharp pain [26]. In a large-scale U.S. study, women received 25% less opioids than men following appendectomies, highlighting a broader trend of undertreatment [27].

Qualitative research has further shown that unconscious gender biases among healthcare providers contribute to the undertreatment of women’s pain. Women’s reports of pain are sometimes interpreted as emotional or psychosomatic rather than clinical, particularly in invasive procedures [13]. This bias is compounded by the fact that many clinical protocols are based on research conducted primarily in male populations and fail to account for physiological differences between sexes, including different responses to analgesics such as morphin [27].

These disparities are not limited to in-hospital care. A study by Wimblish et al. (2022) [28] found that emergency medical service (EMS) providers in Wyoming administered opioids less frequently to women, as well as to Hispanic and American Indian/Alaska Native patients, compared with White male patients. These findings emphasize the need to address both gender and ethnic disparities in prehospital pain management.

Encouragingly, some healthcare systems have started to implement innovative, gender-sensitive interventions. These include staff training on implicit bias, the development of gender-adapted treatment protocols, and the integration of alternative methods such as guided imagery, therapeutic touch, and patient-centered communication. Preliminary results from these initiatives suggest improvements in women’s satisfaction and trust toward medical professionals [13] (Table 1).

## 4. Discussion

The current review provides compelling evidence for the persistent gender disparities in pain assessment and management, particularly in the context of invasive gynecological procedures. Across the studies analyzed, women were consistently found to receive less adequate analgesia than men, and their reports of pain were more frequently underestimated or dismissed by healthcare providers. This ongoing trend reflects a deeply rooted gender bias in medicine, whereby women’s pain is often interpreted through a psychological or emotional lens rather than being acknowledged as a valid clinical symptom. As noted by Samulowitz et al. (2018) [13], this tendency contributes to a diminished standard of care and reinforces inequality in health outcomes between genders.

Several intersecting factors contribute to these disparities. On an individual level, healthcare providers may unconsciously apply gender-based stereotypes—such as assumptions that women are more emotional, have lower pain thresholds, or tend to exaggerate their symptoms. These beliefs, though often implicit, can significantly influence clinical decision-making. They may result in reduced empathy, skepticism toward patients’ self-reports, and a delayed or insufficient therapeutic response. Such attitudes not only compromise the quality of care but may also lead to the normalization of women’s suffering, particularly when expressed during procedures that are traditionally under-medicated.

At the systemic level, the lack of standardized, gender-sensitive clinical guidelines for pain management, especially in routine yet invasive gynecological procedures, further exacerbates these issues. Procedures such as hysteroscopies and intrauterine device (IUD) insertions are still frequently performed with little or no analgesia, despite substantial evidence indicating that these procedures cause significant discomfort and pain. For example, Asgari et al. (2017) [24] demonstrated that local anesthesia effectively reduces pain during hysteroscopy, yet this practice has not been universally adopted, reflecting both institutional inertia and a lack of prioritization of women’s pain.

The implications of undertreated pain in women are profound. Beyond the immediate physical discomfort, the experience of having one’s pain dismissed or inadequately addressed can erode trust in healthcare providers and systems. Women who repeatedly feel invalidated may become reluctant to seek future care, potentially delaying diagnoses and worsening health outcomes. In this sense, pain undertreatment becomes not only a clinical issue but also a public health concern. Furthermore, these disparities raise critical ethical questions regarding autonomy, dignity, and the right to equitable and evidence-based medical treatment.

To address this multifaceted problem, educational and institutional reforms are urgently needed. Medical curricula must be updated to include content on sex and gender differences in pain perception, expression, and treatment efficacy. Moreover, training should incorporate strategies for recognizing and mitigating unconscious bias, as well as fostering empathetic and culturally sensitive communication. As emphasized by Samulowitz et al. (2018) [13], awareness of implicit bias—even when it cannot be fully eliminated—can lead to more equitable and respectful patient interactions.

In addition to reforming provider education, greater involvement of women in the design and evaluation of healthcare services is essential. When patients participate in shaping clinical protocols, assessment tools, and pain management strategies, the resulting systems are more likely to reflect their real-world needs and experiences. Creating clinical environments where women feel heard, respected, and believed is foundational to improving both satisfaction and treatment adherence.

Technological innovations may offer further opportunities to reduce bias and enhance clinical accuracy. For instance, AI-assisted tools or wearable biosensors could facilitate more objective, real-time pain assessments, potentially alerting providers to inconsistencies between reported symptoms and physiological indicators. However, such technologies must be integrated with care, ensuring that they support, not replace, empathy provider–patient relationships and that they are deployed ethically and equitably across populations.

Finally, future research must continue to explore the physiological, psychological, and sociocultural mechanisms that underlie gender differences in pain experience and response. Many clinical trials still lack adequate representation of women or fail to conduct sex-based analyses, leading to gaps in the evidence base that undermine the development of gender-sensitive care. Addressing these research deficiencies is critical for informing clinical practice and guiding policy changes aimed at reducing disparities.

The limitations of this review must be acknowledged. First, the literature search was limited to English-language databases and included only articles published in English and Hebrew. This may have resulted in the exclusion of relevant studies published in other languages and introduced a potential language and publication bias. Second, the review primarily focused on gynecological procedures such as IUD insertions and hysteroscopies. This may have limited the generalizability of findings to other medical contexts where women experience similar disparities in pain management. Future research should consider broader procedural categories to ensure a more comprehensive understanding of gender disparities in clinical pain treatment. Third, although quality appraisal tools were used, the review lacks a detailed critical evaluation of the methodological quality of the included studies. The variability in study design, population, and outcome measures may influence the interpretation of findings. Finally, due to the heterogeneity of the included studies, a meta-analysis was not feasible. The reliance on narrative synthesis limits the generalizability of the findings and highlights the need for future systematic reviews and meta-analyses to establish stronger evidence-based conclusions.

Based on the findings of this review, several actionable recommendations can be proposed to improve pain management for women during medical procedures. First, healthcare providers should receive structured training in recognizing and mitigating implicit gender bias, with particular attention to pain perception and treatment. Second, standardized, evidence-based, gender-sensitive pain management protocols should be developed and implemented, particularly for procedures where undertreatment is common. Third, health policy frameworks should support the integration of patient voices—especially women’s lived experiences—in the development of pain assessment tools and care standards. Finally, future research should aim to include more diverse populations and adopt rigorous, sex-sensitive methodologies to address current evidence gaps and promote equitable pain care.

## 5. Conclusions

This review underscores the persistent gender disparities in pain management, particularly in medical and gynecological contexts. Despite the increasing awareness and growing empirical evidence, women continue to face clinical, cultural, and structural barriers that diminish the legitimacy of their pain reports and compromise their access to effective care. These disparities are rooted in both outdated medical traditions and ongoing institutional gaps, including the absence of gender-specific guidelines and the underrepresentation of women in research.

Closing the gender gap in pain care requires a comprehensive and coordinated response—one that includes educational reform, structural change, and meaningful engagement with women’s voices in healthcare design. It is both a clinical necessity and an ethical obligation. Only through sustained efforts to confront bias, close knowledge gaps, and redesign care systems can we ensure that women’s pain is no longer overlooked, minimized, or inadequately treated.

## Figures and Tables

**Table 1:** Summary of key studies on gender bias in pain management and recommendations for improving care.

Author (Year)	Procedure/Treatment	Key Findings	Recommendations/ Interventions
Samulowitz et al. (2018) [13]	Pain management in ER and clinics	Women receive fewer pain medications than men despite similar pain levels.	Staff training, awareness of gender biases
Guzikevits et al. (2024) [10]	Emergency department (ED) pain management	Analysis of 21,851 ED patient records from two countries revealed a consistent sex bias: women were less likely to receive pain medications than men, even after adjusting for pain severity and other variables. Nurses were 10% less likely to record women’s pain scores, and women spent 30 min longer in the ED. Clinical vignettes confirmed bias in pain intensity judgments by nurses.	Raise awareness of implicit gender bias in clinical decision-making; implement policy changes to ensure equal pain treatment; provide training for healthcare providers
Estevez et al. (2024) [23]	IUD insertion	Only 30% of physicians offer anesthesia; 70% of women report moderate to severe pain.	Use of cervical anesthesia
Asgari et al. (2017) [24]	Cervical anesthesia	Anesthesia significantly reduces pain perception.	Systematic adoption of anesthesia protocols
De Silva PM et al. (2020) [25]	Diagnostic hysteroscopy without anesthesia	Pain levels 7–9 out of 10, especially among women with anxiety.	Holistic treatment, including anxiety management
Dougher et al. (2019) [26]	Cystoscopy	About 40% of clinics provide no pain relief beyond relaxation advice.	Improve pain management in procedures
Serdarevic et al. (2017) [27]	Prescription opioid use	Women are more likely than men to be prescribed opioids, use them chronically, and receive higher doses; women are also at increased risk of misuse.	Implement sex-specific guidelines for opioid prescribing; enhance monitoring and education to reduce misuse and improve pain outcomes
Wimblish et al. (2022) [28]	Prehospital pain management by EM	EMS providers administered opioids significantly less often to women and to Hispanic and American Indian/Alaska Native patients compared with White and male patients.	Address racial, ethnic, and gender disparities in EMS opioid administration through training and protocol standardization

This table summarizes key studies addressing gender disparities in pain management. It includes clinical settings, key findings, and suggested interventions. Abbreviations: ED = Emergency Department; EMS = Emergency Medical Services.

## Data Availability

Not applicable. No new data were created or analyzed in this study. Data sharing is not applicable to this article.
